# 1398. Successful Treatment of primary and refractory Echinococcus (Hydatid Cyst Disease) with Mefloquine and Mebendazole

**DOI:** 10.1093/ofid/ofad500.1235

**Published:** 2023-11-27

**Authors:** James D Richardson, Venus Skeen

**Affiliations:** Texas Tech School of Medicine, Midland, Texas; Midland Memorial Health, Midland, Texas

## Abstract

**Background:**

Treatment regimens for relapsed or refractory Echinococcus (Hydatid cyst disease) are not well defined. Because seropositivity for Echinococcus disease is unpredictable, insurance coverage for expensive regimens are often denied. Improved, effective regimens are needed. Recent laboratory studies in rat model of Echinococcus showed that Mefloquine added to regimens of Mebendazole were effective.

Hydatid cyst pretreatment.

**Figure:**
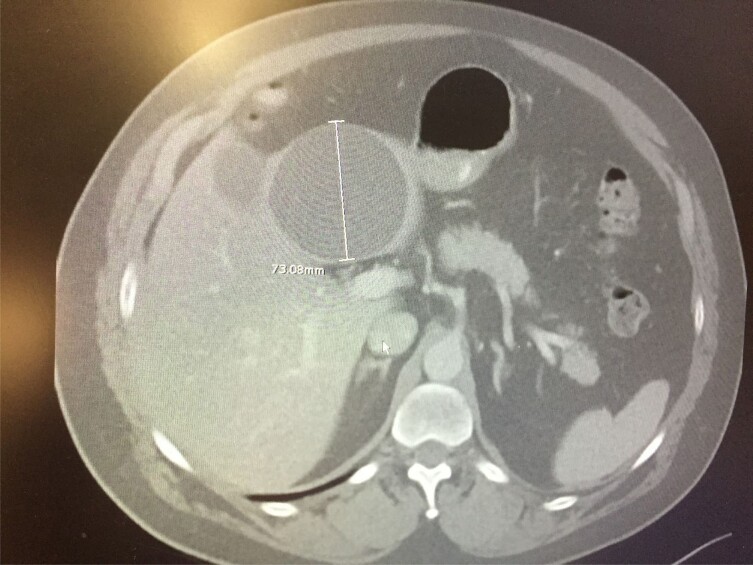

Post treatment liver

**Figure:**
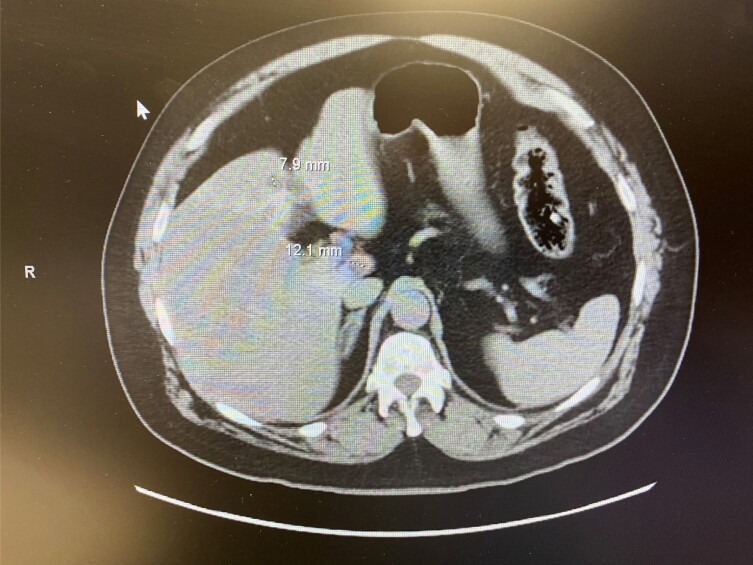

Post treatment CT, 2 years after Mefloquine and Mebendazole treatment.

**Methods:**

Described herein, are three cases of patients treated with combination of Mefloquine and Mebendazole. The initial case was a 56 yo farmer who failed standard therapy with Mebendazole, then PAIRS therapy with Mebendazole. His case was determined to be refractory to therapy. Serologic Echinococcus testing was negative, but CT scan showed liver cyst full of Echinococcus progeny. He was placed on Mebendazole bid for one month with mefloquine 250 mg tiw for one month. He was monitored with weekly liver enzyme tests and baseline EKG. Repeat CT scan at 2 months and 2 years show 2 small stable cysts remaining. Since the first treatment, two other primary cases (one liver and one cerebral) were treated with equally effective results. Patients were informed that this regimen was using medicine that was used to treat parasites, but the combination of the two agents had not been studied extensively together.

**Results:**

Three cases of Echinococcus have been treated with combination of Mebendazole and Mefloquine. All have shown clinical cure. Minor elevations of hepatic enzymes were observed with one case, but regimen was not discontinued. No arrhythmias were seen with the regimen. The refractory case is stable at 2 years. The 2 primary cases are stable at 6 months at follow up.

**Conclusion:**

The combination of Mefloquine and Mebendazole was a well tolerated and effective therapy for both primary and refractory Echinococcus disease. It will be difficult to recruit and study this disease due to the paucity of cases in the US. More study of this disease is needed, but this limited study suggests that this combination regimen of mefloquine and Mebendazole has the potential to be a new standard of care. Moreover, this treatment regimen shortens therapy from 3 months to one month.

**Disclosures:**

**All Authors**: No reported disclosures

